# Effects of Chitosan and Cellulose Derivatives on Sodium Carboxymethyl Cellulose-Based Films: A Study of Rheological Properties of Film-Forming Solutions

**DOI:** 10.3390/molecules28135211

**Published:** 2023-07-04

**Authors:** Huatong Zhang, Shunjie Su, Shuxia Liu, Congde Qiao, Enhua Wang, Hua Chen, Cangheng Zhang, Xiaodeng Yang, Tianduo Li

**Affiliations:** 1 Shandong Key Laboratory of Molecular Engineering, State Key Laboratory of Biobased Material and Green Papermaking, Qilu University of Technology (Shandong Academy of Sciences), Jinan 250353, China; 10431200211@stu.qlu.edu.cn (H.Z.); shunjiesu@163.com (S.S.); cdqiao@qlu.edu.cn (C.Q.); weallydia@163.com (E.W.); canghengzhang@163.com (C.Z.); 2 Shandong Provincial Technology Center of Jining Zhongyin Electrochemical Co., Ltd., Jining 272500, China; 329-5liu@163.com; 3 Interventional Department of Shandong Provincial Cancer Hospital Affiliated to Shandong First Medical University, Jinan 250117, China

**Keywords:** carboxymethyl chitosan, chitosan derivative, cellulose derivative, film-forming solution, rheological properties

## Abstract

Bio-based packaging materials and efficient drug delivery systems have garnered attention in recent years. Among the soluble cellulose derivatives, carboxymethyl cellulose (CMC) stands out as a promising candidate due to its biocompatibility, biodegradability, and wide resources. However, CMC-based films have limited mechanical properties, which hinders their widespread application. This paper aims to address this issue by exploring the molecular interactions between CMC and various additives with different molecular structures, using the rheological method. The additives include O-carboxymethylated chitosan (O-CMCh), N-2-hydroxypropyl-3-trimethylammonium-O-carboxymethyl chitosan (HTCMCh), hydroxypropyltrimethyl ammonium chloride chitosan (HACC), cellulose nanocrystals (CNC), and cellulose nanofibers (CNF). By investigating the rheological properties of film-forming solutions, we aimed to elucidate the influencing mechanisms of the additives on CMC-based films at the molecular level. Various factors affecting rheological properties, such as molecular structure, additive concentration, and temperature, were examined. The results revealed that the interactions between CMC and the additives were dependent on the charge of the additives. Electrostatic interactions were observed for HACC and HTCMCh, while O-CMCh, CNC, and CNF primarily interacted through hydrogen bonds. Based on these rheological properties, several systems were selected to prepare the films, which exhibited excellent transparency, wettability, mechanical properties, biodegradability, and absence of cytotoxicity. The desirable characteristics of these selected films demonstrated the strong biocompatibility between CMC and chitosan and cellulose derivatives. This study offers insights into the preparation of CMC-based food packaging materials with specific properties.

## 1. Introduction

The growing concern for sustainable development has led to increased interest in bio-based packaging materials, such as commodity packaging materials and drug carriers [1,2]. Among these materials, cellulose has emerged as a favorable option due to its wide availability, biodegradability, non-cytotoxicity, thermostability, and chemical stability [3]. However, the water-insolubility of cellulose greatly limits its practical applications. To overcome this limitation, cellulose is often modified to enhance it water solubility. Sodium carboxymethyl cellulose (CMC) is a water-soluble derivative commonly used in food packaging due to its favorable properties inherited from cellulose, in addition to its water solubility [4].

While CMC films have shown promise as food packaging materials, they often lack sufficient mechanical properties [5]. To address this issue, researchers have explored the incorporation of polymers or polymer-based nanomaterials, such as chitosan and cellulose derivatives, to enhance the mechanical properties and thermodynamic stability of CMC films [6,7,8].

Chitosan, a natural linear polymeric amino sugar with repeating units of amino and hydroxyl groups, has attracted attention for its biocompatibility, biodegradability, and non-toxicity [9,10,11,12]. The electrostatic interaction and hydrogen bonding between chitosan and CMC can play a key role in moderating the properties of chitosan–cellulose materials. Rheological tests are widely used to analyze intermolecular interactions and predict material properties [2,13].

The rheological properties of the polymeric solutions are influenced by various factors, such as molecular weight ($M_{w}$), molecular weight distribution, degree of substitution (DS), and polymer concentration (C_CMC_). For example, CMC solutions exhibit shear thinning behavior, Newtonian fluid behavior, and shear thickening behavior as the $M_{w}$ increases from 9 × 10^4^ to 25 × 10^4^ and 70 × 10^4^ while at fixed concentrations and DS [14]. The CMC solutions also exhibit Newtonian fluid behavior, shear thinning, and shear thickening behaviors as the concentration increases to 0.5, 2.0, and 3.0 g/L, respectively [15]. The apparent viscosity of the CMC solutions increases with increasing $M_{w}$, DS, and C_CMC_. CMC with a large $M_{w}$ shows a more pronounced effect on rheological properties [16].

To the best of our knowledge, there is a lack of research on the systematic preparation of CMC-based films according to the properties of film-forming solutions. In the current paper, the rheological properties of CMC-based film-forming solutions are studied and the effects of chitosan and cellulose derivatives are compared. The chitosan derivatives include O-carboxymethylated chitosan (O-CMCh), hydroxypropyltrimethyl ammonium chloride chitosan (HACC), and N-2-hydroxypropyl-3-trimethylammonium-O-carboxymethyl chitosan (HTCMCh), which are anionic, cationic, and amphiphilic chitosan derivatives, respectively. The cellulose derivatives consist of cellulose nanocrystals (CNC) and cellulose nanofibers (CNF). The molecular structure, content of the derivatives, and temperature are investigated. Based on the rheological results, selected CMC-based films are prepared and their properties, including wettability, transparency, hydrophilicity, mechanical properties, degradation, and toxicity, are evaluated. This work provides valuable knowledge for the development of film-forming solutions for the targeted preparation of food packaging materials.

## 2. Results and Discussion

### 2.1. Effect of Chitosan and Cellulose Derivative Structures on Rheological Properties

To investigate the effect of the molecular structure on the rheological properties of CMC-based solutions, chitosan derivatives of HTCMCh, O-CMCh, and HACC as well as cellulose derivatives of CNC and CNF with a mass content of 1% were selected.

**Figure 1:**
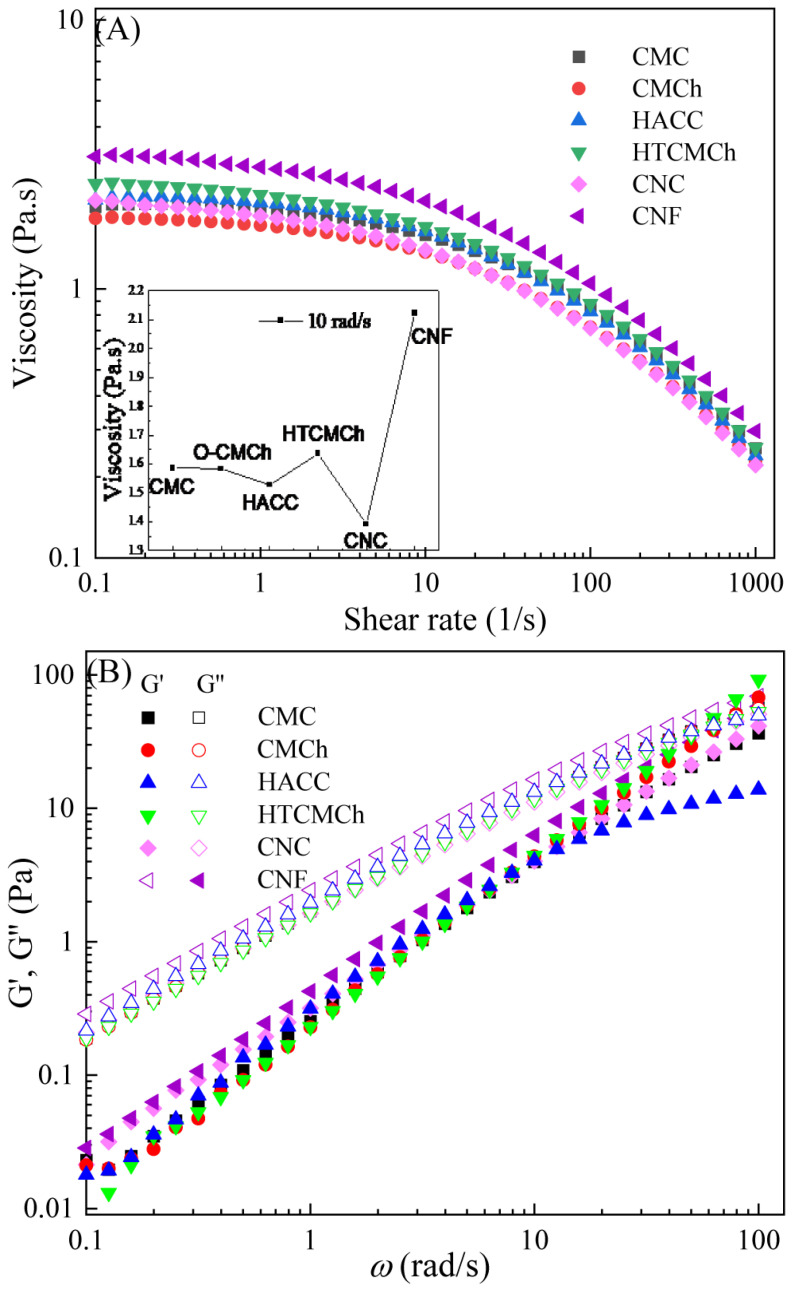
Apparent viscosity (**A**) and viscoelastic moduli (**B**) of CMC-based film-forming solutions at 25 °C. The insert in A included the viscosities of the film-forming solutions at a shear rate of 1 s^−1^. The content of HTCMCh, O-CMCh, HACC, CNC, and CNF was 1%.

The CMC solution exhibited Newtonian behavior at shear frequencies below 1 s^−1^, and shear thinning behavior at frequencies above 1 s^−1^, as shown in Figure 1A. The Newtonian behavior can be attributed to the hydrogen bonding and entanglement network between CMC molecular chains. As the shear rate increased, the hydrogen bonding and entanglement network were disrupted, resulting in shear thinning behavior [7,15]. Utilizing this shear thinning behavior, it is possible to control the size distribution of polymer or colloid particles in solution, thereby achieving the preparation of membrane materials with a regular morphology and excellent performance.

The addition of HTCMCh, O-CMCh, HACC, CNC, and CNF caused changes in the viscosity of the CMC solution. However, it did not affect the Newtonian shear thinning behavior of the solutions (Figure 1A). The chitosan derivatives had a limited influence on the viscosity of the CMC solution, while the cellulose derivatives had a more significant impact. CNC decreased the viscosity of the CMC solution, while CNF significantly increased the viscosity (as shown in the insert in Figure 1A). The CMC/CNC_1%_ system exhibited the lowest viscosity. There are three possible explanations for this phenomenon. Firstly, CNC particles have a rod-like shape with low aspect ratios, allowing for better dispersion within the CMC matrix, which reduces interchain entanglements and results in lower viscosity. Secondly, CNC particles can interact with CMC through hydrogen bonding, causing a change in the conformation of CMC chain and reducing the viscosity of the solution. Lastly, the addition of CNC could also affect the hydrodynamic volume and orientation of CMC chains, leading to a decrease in viscosity. CNF increases the viscosity of the CMC film-forming solution, thereby improving the film-forming rate and quality. CNF displayed the greatest increase in viscosity, followed by HTCMCh, while CNC showed the least enhancement. This can be attributed to the largest aspect ratio of CNF and the presence of a large number of hydrogen bonds. On the other hand, CNC had a low aspect ratio [17]. It is worth noting that the addition of HTCMCh resulted in an increase in viscosity, while O-CMCh led to a slight decrease. This can be attributed to the different interactions between O-CMCh, HTCMCh, and CMC. The repulsion between CMC and O-CMCh can weaken the hydrogen bonds between CMC molecules, resulting in a decrease in viscosity. HTCMCh, being an amphiphilic chitosan derivative, interacts with the carboxyl groups in CMC through quaternary ammonium salt groups, forming entanglements and increasing the viscosity of the solution [18]. An increase in viscosity could result in an uneven coating of the film-forming solution, making it challenging to form a smooth film.

The relationship between the shear rate and shear stress of CMC film-forming solutions was analyzed and fitted using a power-law model (Equation (1)).

(1) $$\sigma =k_{PL}{\dot{\gamma }}^{n}$$

where σ, $k_{PL}$, $\dot{\gamma }$ and *n* represent the shearing stress, consistency coefficient, shear rate, and power-law exponent, respectively. If the value of *n* is less than one, the solution exhibits shear thinning behavior.

The fitted parameters (Table 1) were in agreement with the experimental data, confirming the shear thinning behavior of the film-forming solutions as all the power-law exponent values were less than one. In terms of the consistency coefficient, the viscosity at 1 s^−1^ in the CMC/CNF_1%_ system was the highest, while that of the CMC/CNC_1%_ system was the lowest. Film-forming solutions with lower viscosities can easily fill the micropores and gaps within the film coating, thereby preventing the entry of oxygen, water, and harmful substances, ultimately enhancing the antioxidant performance of the film layer.

The viscoelastic moduli of CMC and CMC/additive_1%_ solutions are shown in Figure 1B, with the storage moduli (G″) being higher than the elastic moduli (G′). Previous studies have suggested that the viscoelastic properties of CMC solutions are primarily dependent on the CMC concentration [19]. Benchabane et al. [20] also observed that CMC solutions displayed viscous properties at concentrations of 1 wt% and 3 wt%. While additives can alter the properties of solutions, they generally do not affect the fundamental characteristics, such as viscosity and elastic modulus [21]. These two parameters are important for describing the flow and deformation behavior of liquids and can be linked to the intermolecular interactions within the liquid [22]. At an angular frequency of 50.12 rad/s, the elastic moduli of the CMC/HTCMCh_1%_ and CMC/O-CMCh_1%_ film-forming solutions exceeded their viscous counterparts, indicating intermolecular interactions. During the formation of the liquid film, the molecules need to overcome surface tension and withstand external tensile and shear forces. Therefore, they must possess sufficient deformability and fluidity. If the liquid molecules need to overcome a relatively high energy storage modulus, more energy consumption is required for deformation and flow, resulting in a slower film-forming rate and potentially hindering the film-forming process.

### 2.2. Effect of Derivative Concentration on Rheological Properties

**Figure 2:**
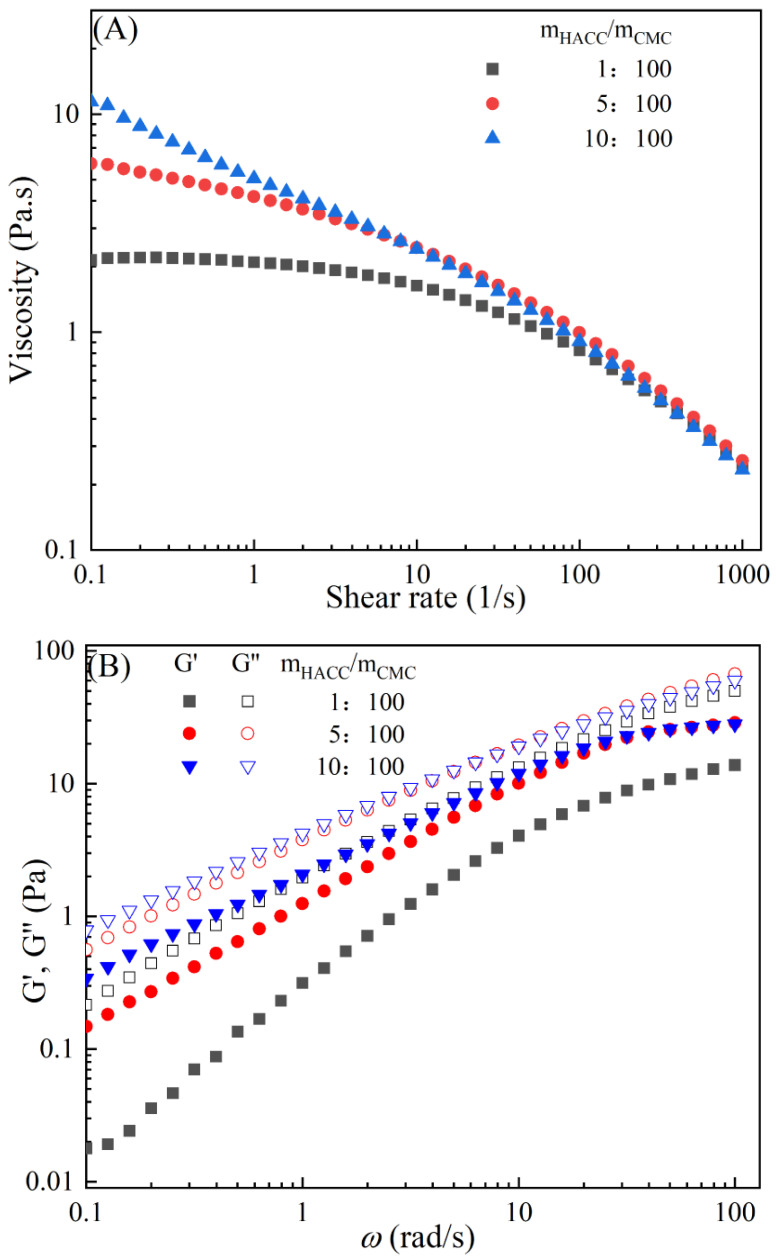

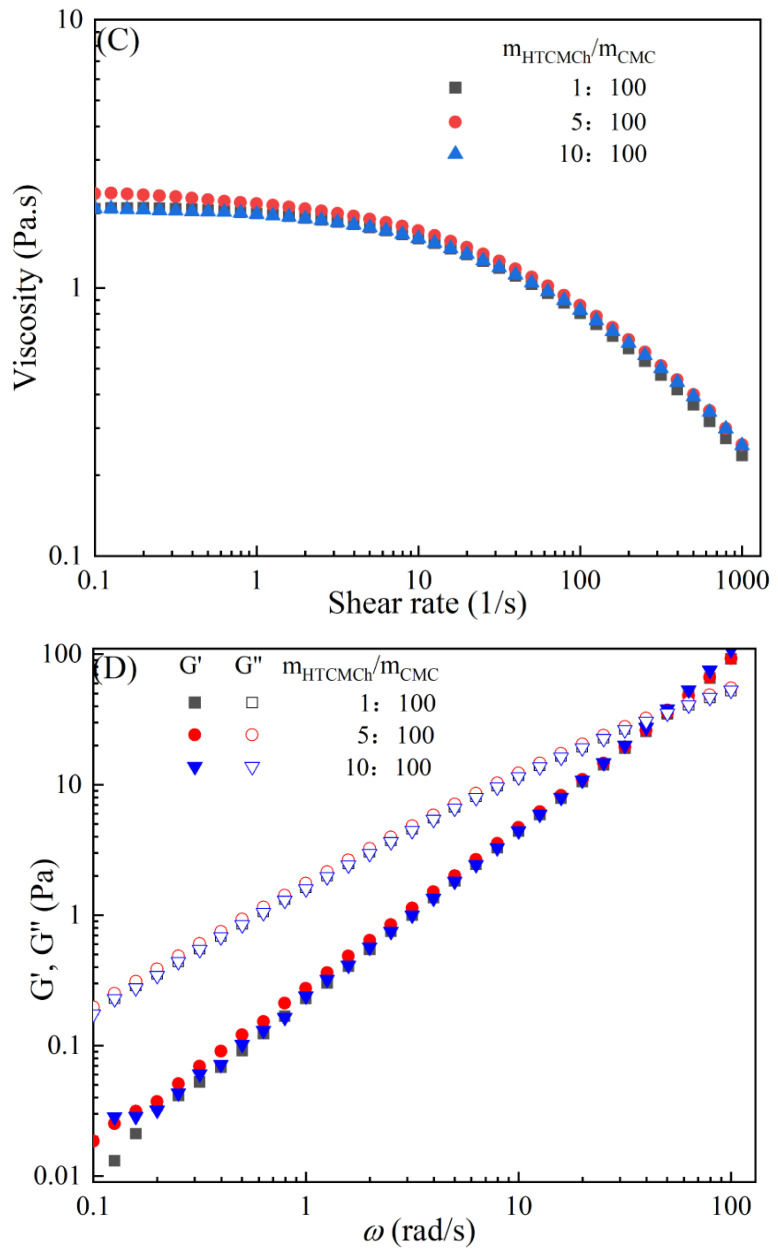

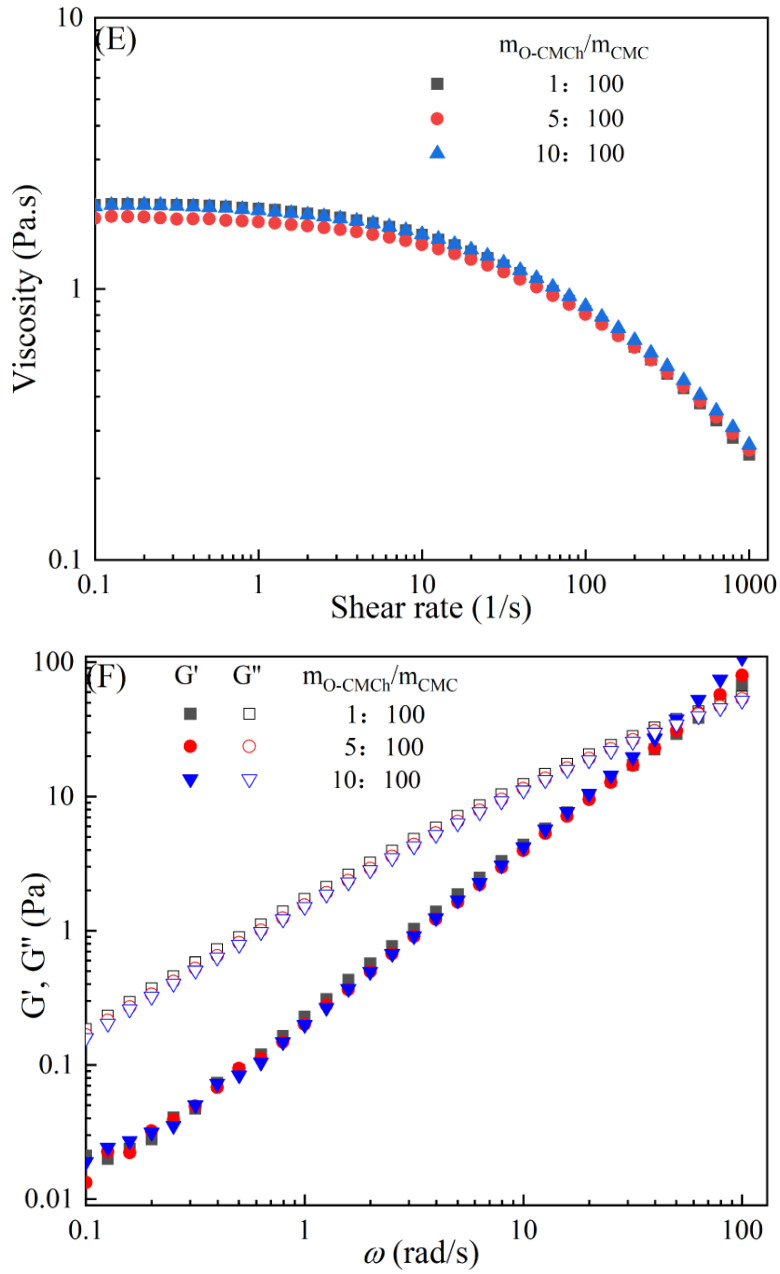

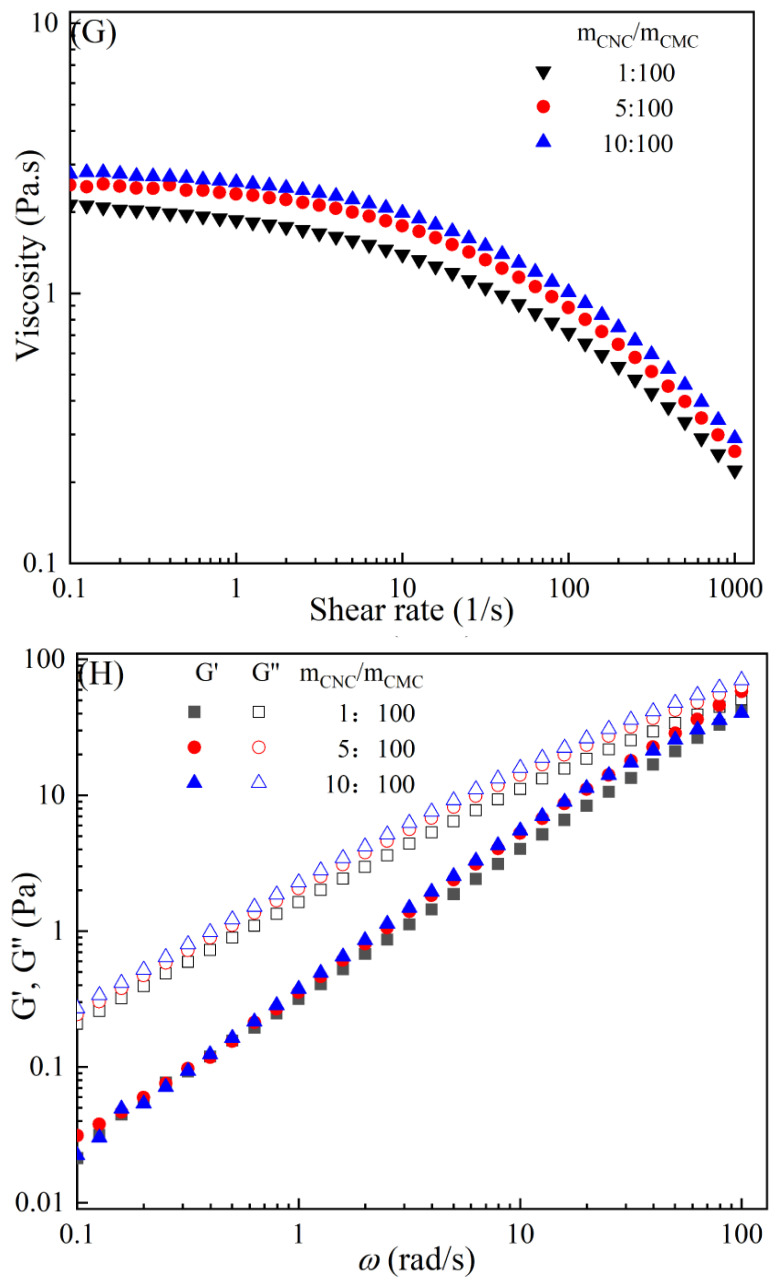

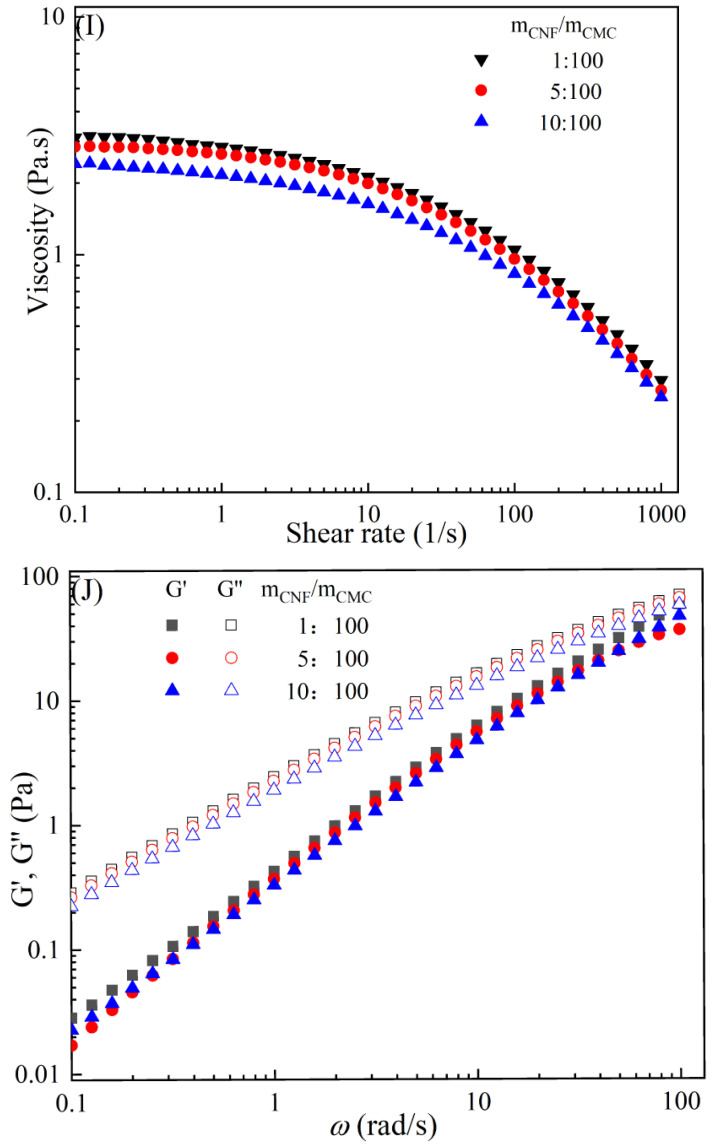
Apparent viscosity (**A**,**C**,**E**,**G**,**I**) and viscoelastic moduli (**B**,**D**,**F**,**H**,**J**) of CMC-based film-forming solutions with different amounts of additives at 25 °C.

The rheological behaviors of CMC-based film-forming solutions with different contents of chitosan and cellulose derivatives are shown in Figure 2. The concentration of HACC showed a significant influence on the viscosity of CMC/HACC film-forming solutions within a shear rate of 0.1–100 s^−1^. As the HACC concentration increased from 1% to 10%, the viscosity of the solution rapidly increased from 2.09 to 5.07 Pa·s (Figure 2A). On the other hand, the contents of CNC, CNF, HTCMCh, and O-CMCh exhibited minimal effects on the rheological properties of film-forming solutions (Figure 2C,E,G,I). HACC, a type of cationic chitosan derivative, can interact with CMC through electrostatic forces and hydrogen bonding, leading to an increase in viscosity. In contrast, O-CMCh, an anionic chitosan derivative, exhibits strong electrostatic repulsion with CMC. HTCMCh, an amphiphilic chitosan derivative, experiences both intra- and inter-molecular electrostatic interactions at high concentrations. These interactions contribute to the increase in viscosity and result in the lowest onset rate of shear thinning behavior among the film-forming solutions [23]. Similar phenomena have been observed in other systems [18,23]. CNC and CNF affect the viscosity of the CMC-based film-forming solution through their physical obstacles and the formation of hydrogen bonds with CMC [24,25].

The viscoelasticity of the CMC/HACC film-forming solution was significantly affected by the HACC content within an angular frequency from 0.1 to 100 rad/s. With an increase in the HACC concentration from 1% to 10%, the storage modulus increased from 4.06 to 11.93 Pa (Figure 2B). This can be attributed to the strong electrostatic interaction between the quaternary ammonium groups in HACC and carboxyl groups in CMC, which enhance the physical interactions between the molecules [26]. Even a small amount of HACC can induce CMC aggregation, strengthening these interactions and enhancing the physical correlation effect [27]. In contrast, the addition of O-CMCh, HTCMCh, CNC, and CNF had little impact on the storage moduli (Figure 2D,F,H,J). This can be attributed to electrostatic repulsion between the macromolecules, as well as the competition between the additives and CMC molecules, leading to the decrease in the intramolecular hydrogen bonding of the CMC molecules [28,29]. The viscoelasticity of a film formed from a solution is related to its thickness and may result in defects such as wrinkles, cracks, and scratches. Additionally, the presence of additives can alter the internal structure and interaction mode of the composite film, influencing its mechanical properties, thermal stability, moisture absorption, and biodegradability. Therefore, it is crucial to carefully consider the type and dosage of additives in the design of CMC composite films to optimize the film-forming process and the performance of the final product.

### 2.3. Effect of Temperature on Rheological Properties

**Figure 3:**
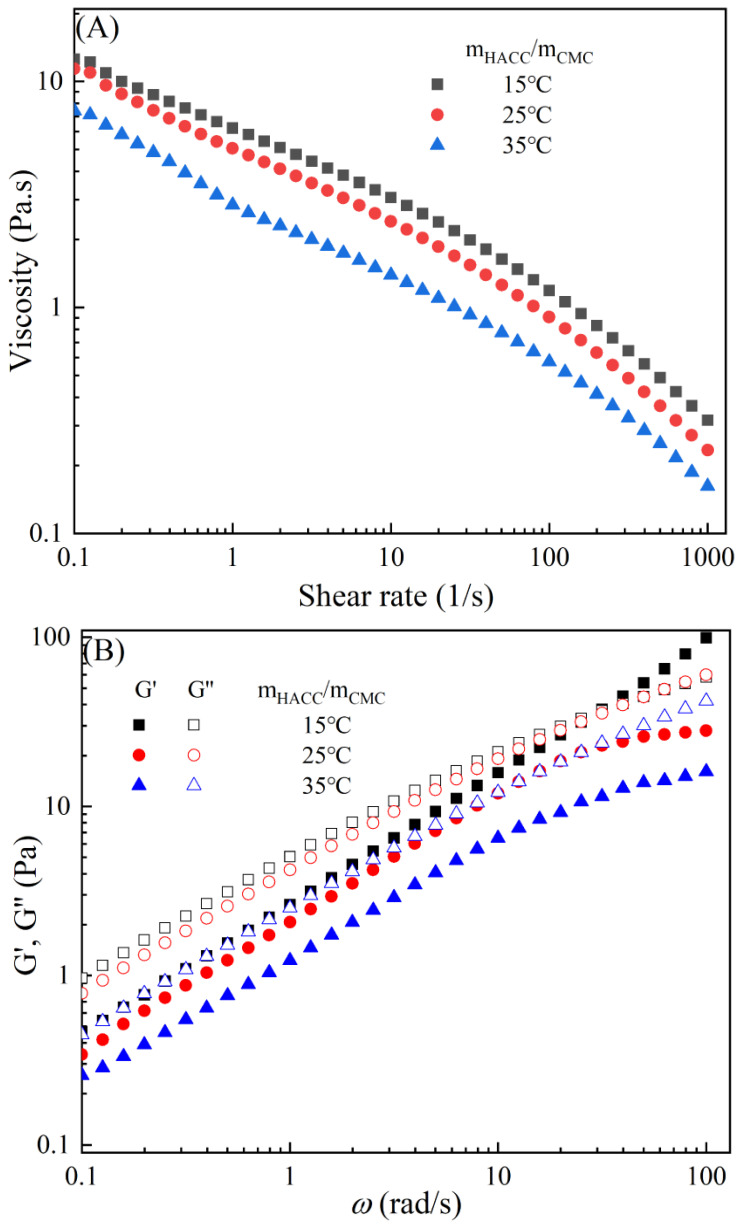

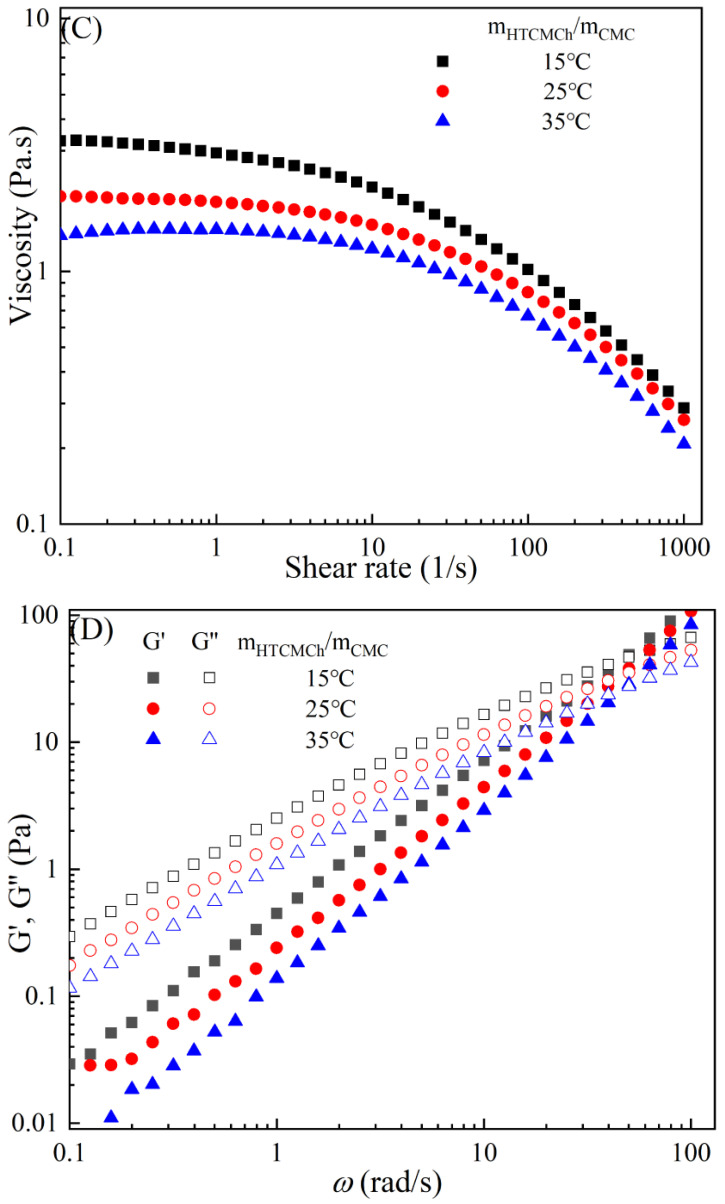

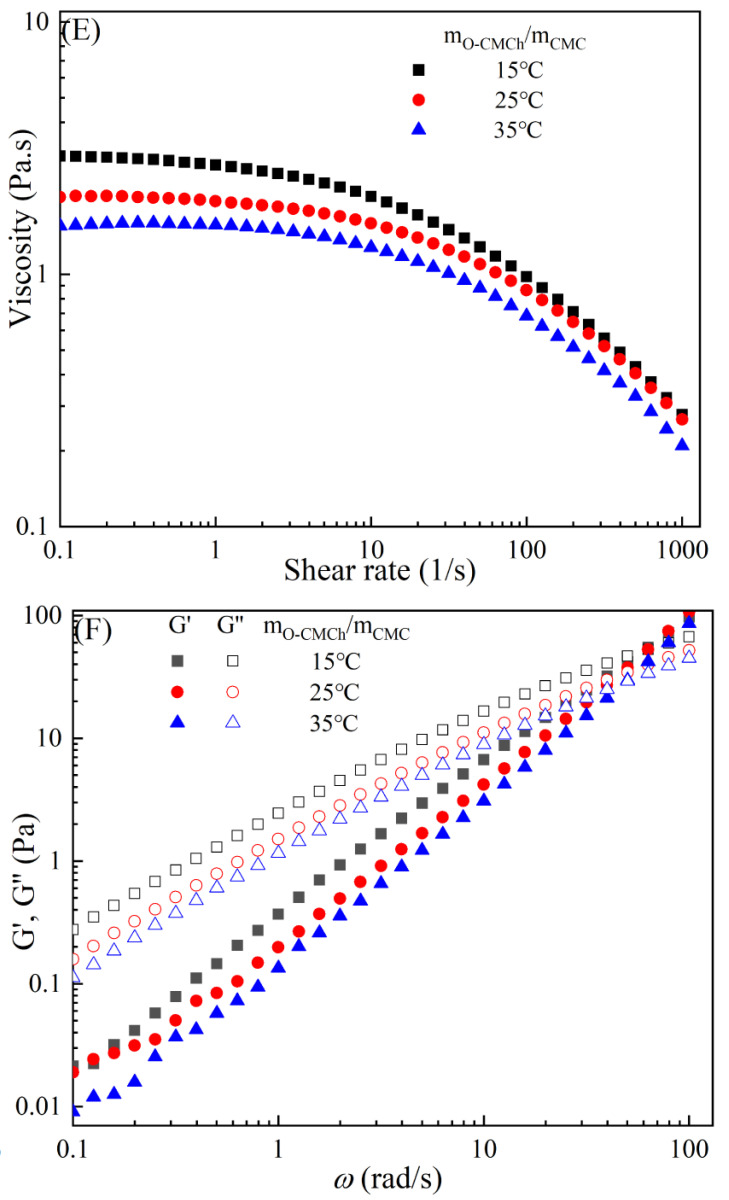

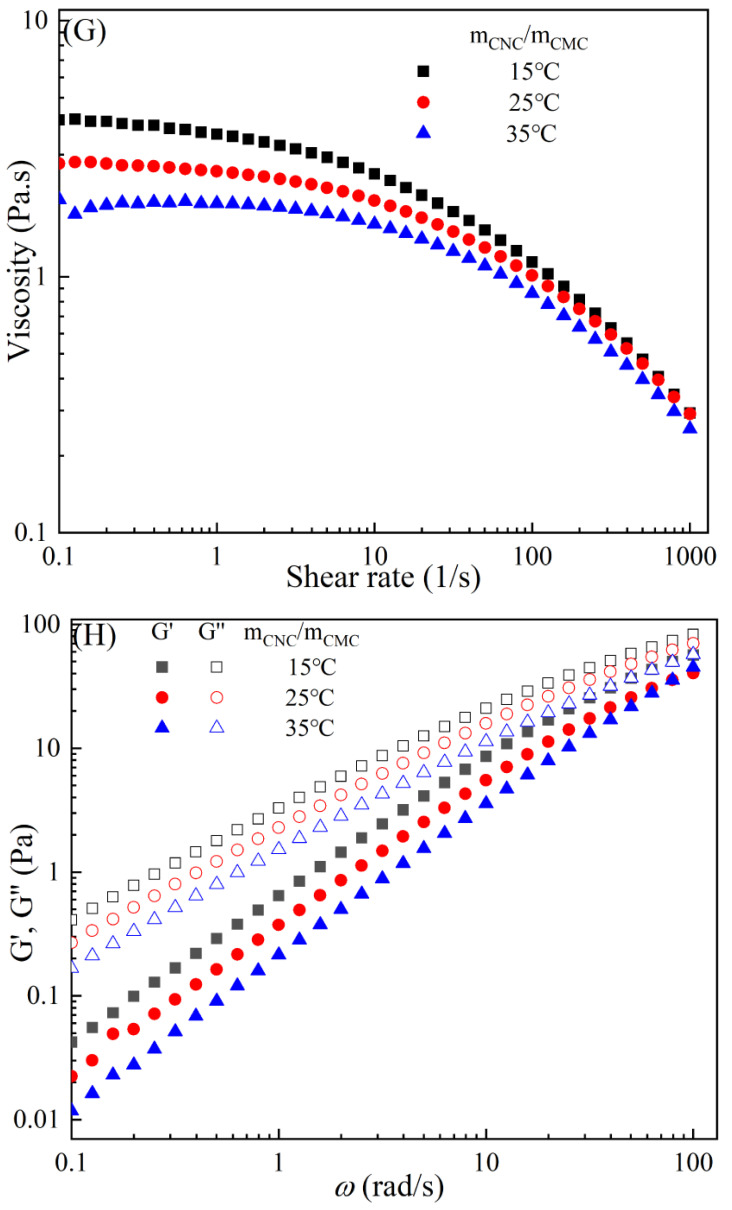

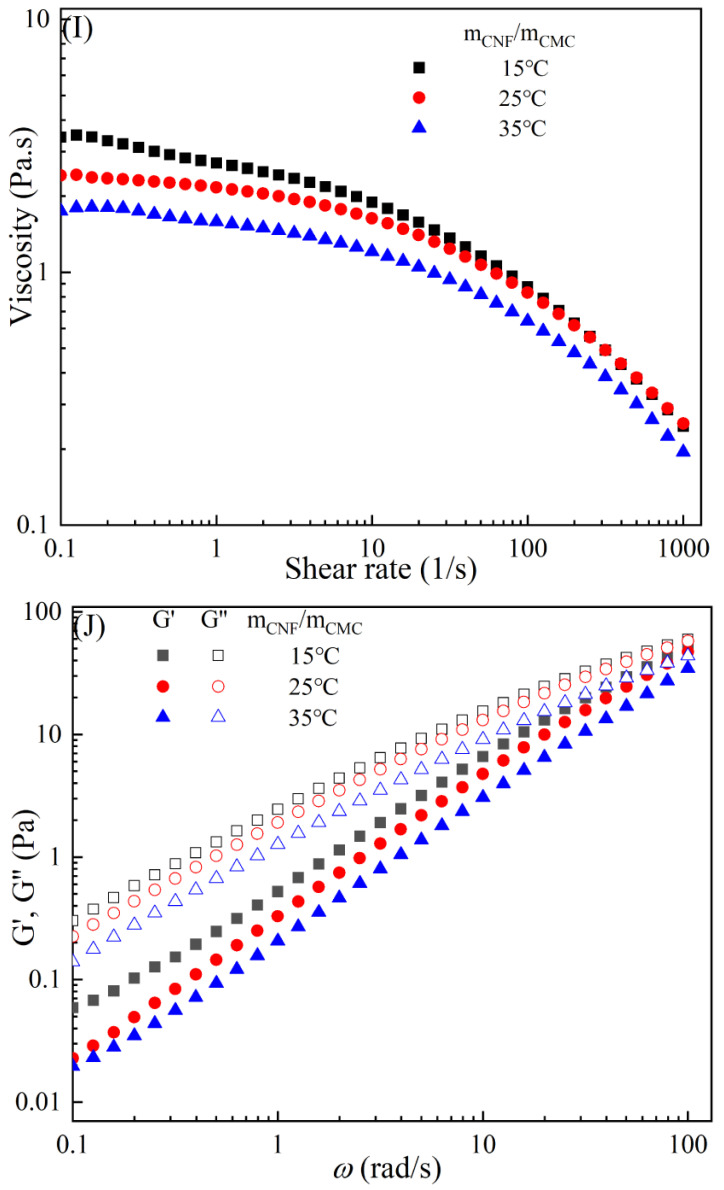
Apparent viscosity (**A**,**C**,**E**,**G**,**I**) and viscoelastic moduli (**B**,**D**,**F**,**H**,**J**) of CMC solutions with 10% additives (HACC, HTCMCh, O-CMCh, CNC, and CNF) at different temperatures.

Figure 3 demonstrates the typical temperature-dependent behavior of the CMC-based film-forming solutions, where both viscosities and dynamic moduli decrease with increasing temperature [30,31]. The decrease in viscosity can be attributed to an increase in the molecular free volume and a corresponding decrease in intermolecular interactions [32,33]. With the rise in temperature, the average kinetic energy of the molecules increases, resulting in an expansion of the average distance between them and an increase in free volume [34]. This temperature-dependent behavior also influences the viscoelastic properties. At lower temperatures, CMC molecules are primarily in a condensed state, with strong intermolecular interactions leading to a higher storage modulus [35]. However, as the temperature rises, a significant amount of thermal energy is input into the system, promoting molecular vibrations and enhancing molecular freedom for CMC molecules [36]. Consequently, the intermolecular interaction forces weaken, leading to a decrease in the storage modulus of CMC. Additionally, under high-temperature conditions, CMC molecules are more susceptible to rheological instability, contributing to the decrease in the storage modulus [37]. Moreover, different temperatures can cause the chemical degradation of CMC, further influencing changes in the storage modulus [38]. Compared to solutions with high storage modulus, those with low storage modulus exhibit greater plasticity and offer better extrusion and stretching performance for membranes.

As is shown in Table 2, an increase in temperature results in a slight increase in the values of n and a notable decrease in $k_{PL}$ [16]. This indicates that the degree of shear thinning and viscosity decreases with rising temperature. Both shear thinning behavior and viscosity are dependent on intermolecular and intramolecular interactions. The CMC/HACC_10%,15°C_ system exhibits the highest $\eta_{1}$ value, which can be attributed to a strong electrostatic interaction between HACC and CMC. According to Yang et al., the strong interaction between HACC and CMC, coupled with the reorganization of these chains, allows the apparent viscosity of the hybrid system to be maintained even at elevated temperatures [18]. Typically, the viscosity of a film-forming solution decreases with increasing temperature due to a reduction in intermolecular forces and increased molecular activity. However, excessively high temperatures can lead to excessively low viscosity, resulting in film formation failure.

### 2.4. Morphology, Thickness, Whiteness, Transmittance, and Wettability of CMC-Based Films

**Figure 4:**
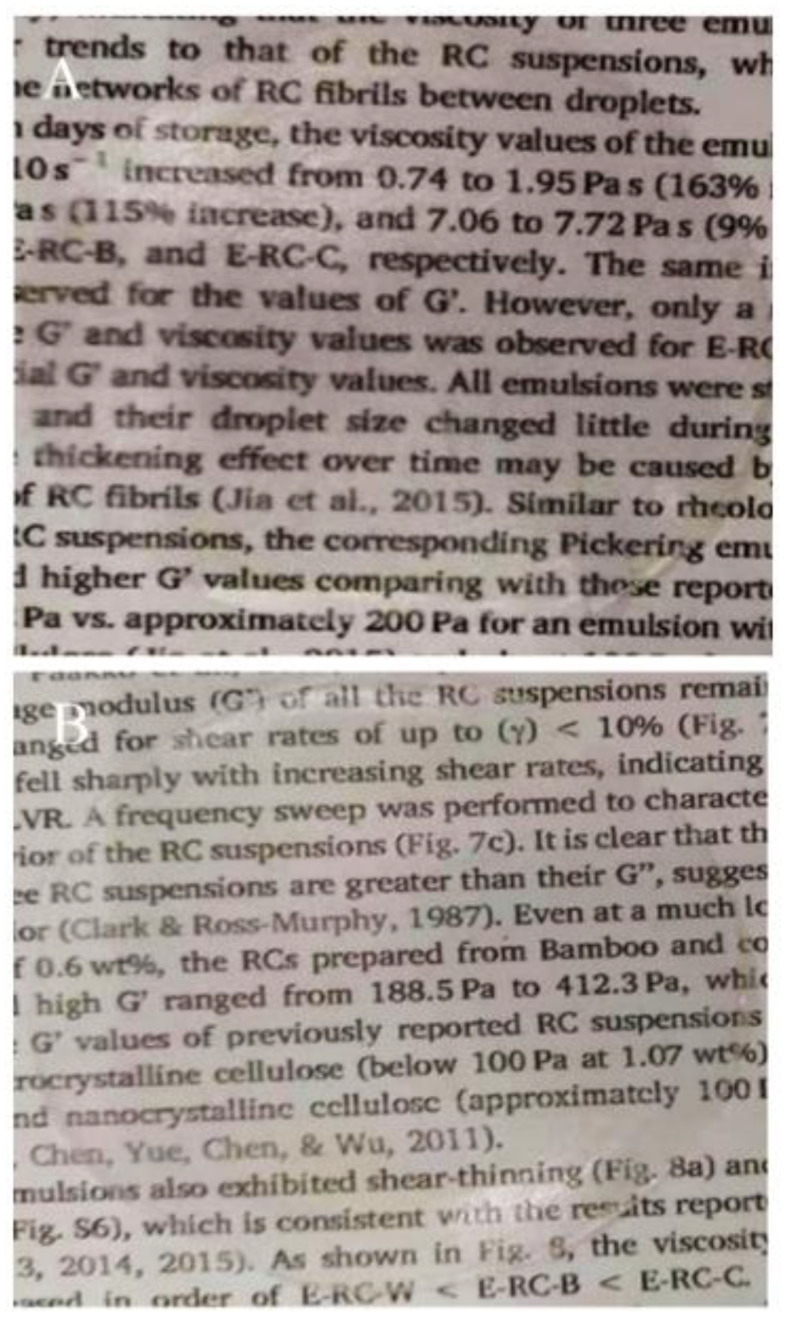

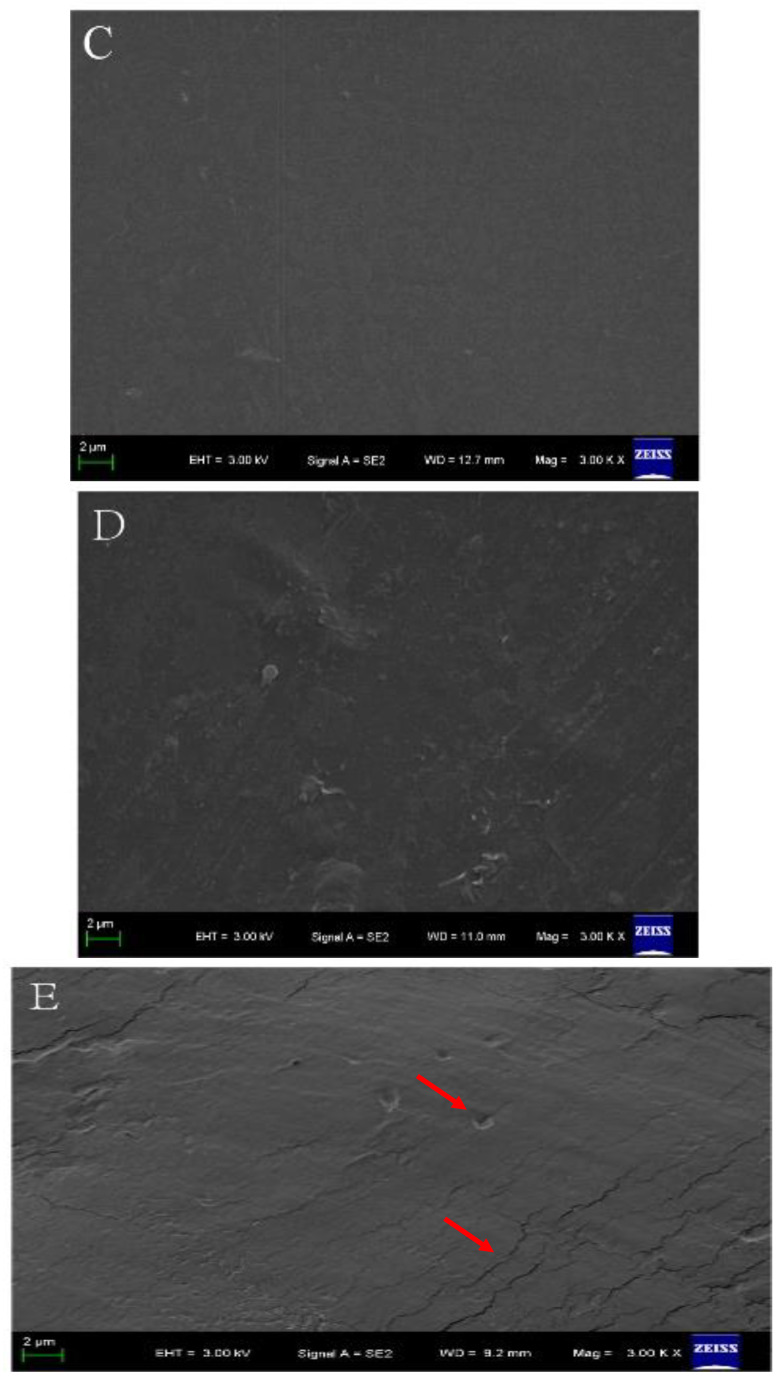
Topography images of CMC/HTCMCh (**A**) and CMC/CNF (**B**), and SEM images of CMC (**C**), CMC/HTCMCh (**D**), and CMC/CNF (**E**). The red arrows show the cracks of the film, and the background contents were authorized by the publisher [41,42].

The film-forming solutions with chitosan and cellulose were used to prepare CMC-based films. Taking the examples of CMC/HTCMCh and CMC/CNF films (Figure 4), it can be observed that these films exhibited good transparency, similar to other polysaccharide-based films. This can be attributed to the excellent compatibility of CMC with chitosan and cellulose derivatives [39]. The pure CMC film displayed a compact, uniform, and smooth surface. The CMC/HTCMCh film exhibited a slightly rough morphology with a few cracks(red arrows in Figure 4), while the CMC/CNF film showed limited cracks on the surface. In a previous study by Zhang and Jin, the microstructure (cross-section) of the CMC/cationic chitosan derivative film showed a rough structure, whereas the CMC/cellulose derivative film had only a limited number of cracks [39,40]. The rough structures were attributed to the aggregates of CMC-cationic chitosan derivatives, resulting from the electrostatic interaction between cationic groups in the chitosan derivative and ${-\mathrm{COO}}^{-}$ groups in CMC [41]. The small number of cracks in the CMC/cellulose derivative film was caused by the hydrogen bonding effect and nanoparticle effect [42]. Additionally, various factors, including film-forming conditions, plasticizers, degree of substitution, the molecular weight of CMC, and the interaction between CMC and additives, can influence the microscopic morphology of CMC-based films [43].

Table 3 presents the thickness of the CMC film. It can be observed that HTCMCh and HACC decreased the film thickness, while HACC, CNC, and CNF increased it.

The film thickness depends on the inherent properties of the film-forming materials, the alignment and interaction between these materials, and the concentration of additives [41]. CMC and HTCMCh are water-soluble and contain groups with opposite charges, resulting in strong electrostatic interactions dominating their arrangement; HACC behaves similarly [44]. Under a high mass ratio of HTCMCh and HACC (e.g., 10%), the electrostatic interactions and hydrogen bonding induce a tight cross-linking between these two macromolecules, leading to a decrease in film thickness. Therefore, an increase in the content of O-CMCh, CNC, and CNF weakens the electrostatic interactions between these molecules, resulting in an increase in film thickness.

The surface properties of the films, including wettability and water vapor permeability, are affected by the intermolecular interaction and arrangement of the macromolecules in the films. The contact angles of the films are listed in Table 3. All of the CMC-based films exhibited hydrophilic properties, with an increase in HTCMCh and HACC contents leading to an increase in contact angles. Conversely, an increase in O-CMCh, CNC, and CNF content resulted in a decrease in contact angles. These changes can be attributed to the different intermolecular interactions as the film thickness varies. These findings are consistent with previous studies on N-(2-hydroxypropyl)-3-trimethylammonium chitosan (HTCC)/CMC film [18] and the tea polyphenol/hydroxypropyl starch film [45].

The whiteness and transmittance of the pure CMC film were 35.34 ± 0.13 and 92.72 ± 0.16, respectively. The addition of chitosan derivatives and cellulose had a minimal effect on the whiteness and transparency of the CMC-based films (Table 3), as confirmed by the optical appearance in Figure 4. This indicates good compatibility between the two biomacromolecules.

### 2.5. Thermogravimetric Properties of CMC-Based Films

**Figure 5:**
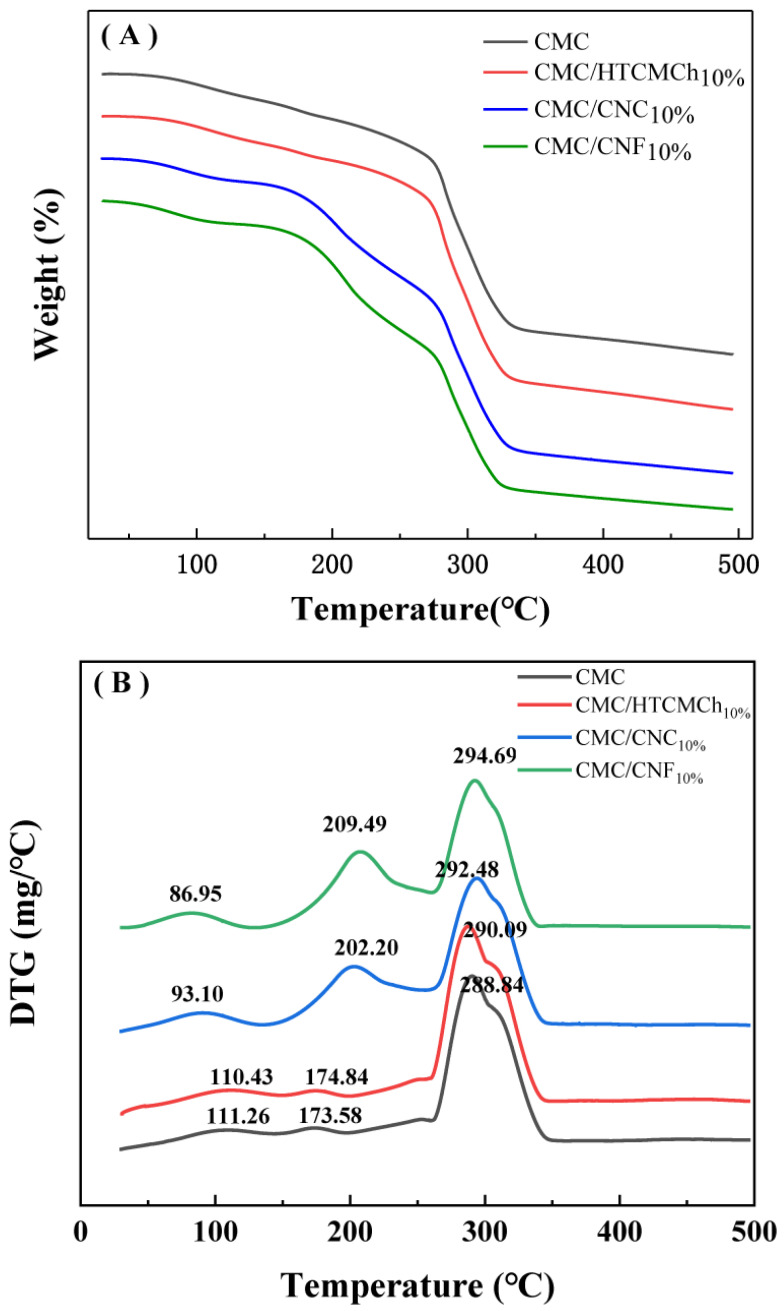
TGA (**A**) and DTG (**B**) cures of CMC-based films.

The intermolecular interactions of the CMC-based films can also be observed in the TGA curves. Figure 5 shows the TGA results of the CMC-based films, with the differential thermogravimetry (DTG) curves being more sensitive. The DTG curve of the pure CMC film exhibited three distinct peaks corresponding to the evaporation of bound water, the dissociation of hydrogen bonds (111 °C), the evaporation of internal water (173 °C), and the decomposition of the cellulose chain (288 °C) [40].

The addition of additives to CMC can lead to improvements in the thermal stability of the composite films through interactions such as hydrogen bonding and electrostatic interactions. However, the destruction of intramolecular and intermolecular hydrogen bonds, as well as the formation of discontinuous structures, can decrease the thermal stability properties of CMC. The presence of 10% HTCMCh had a negligible effect on the thermal stability of the CMC. On the other hand, the incorporation of 10% nanocellulose (CNC and CNF) enhanced the encapsulation of water by the composite film, resulting in a higher evaporation temperature of the encapsulated water. The CMC/CNF_10%_ and CMC/CNC_10%_ films exhibited a higher maximum decomposition temperature (292.48 °C and 294.60 °C, respectively). The inclusion of nanocellulose effectively increased the maximum decomposition temperature of CMC composite films, which can be attributed to a small specific surface area and size of nanocellulose particles [40]. The filling effect of nanocellulose on CMC, along with the strong hydrogen bond with CMC, hindered the relative motion between the molecules, thereby increasing the maximum decomposition temperature of the composite films [42]. The electrostatic interaction between CMC and cationic polymers can also significantly improve the thermodynamic properties of CMC films. For instance, HACC can effectively increase the maximum decomposition temperature of CMC films [18], and HTCMCh can enhance the glass transition temperature of the films from 56.5 °C to 90.2 °C [41]. The electrostatic repulsive interactions between CMC, CNC, and CNF could result in the formation of pinholes and occasional slits in the CMC-based films.

The stronger intermolecular interaction between CMC and 10% CNF, along with the denser structure of the resulting CMC/CNF_10%_ film, contributed to a higher maximum decomposition temperature compared to 10% CNC. This finding is consistent with the rheological results.

### 2.6. Mechanical Properties of CMC-Based Films

Table 3 presents the tensile strength (TS), elongation at break (EB), and Young’s modulus (E) of the CMC-based films. The neat CMC film exhibited a tensile strength of 13.57 MPa and an elongation at break of 44.41 ± 0.61%. The mechanical properties of CMC films were influenced by various factors, including the source of CMC, its molecular mass and distribution, and the film formation conditions [41]. The addition of chitosan and cellulose derivatives resulted in an increase in the tensile strength of the CMC-based films compared to the pure CMC film (Table 3). Among the CMC-based films, CMC/CNF_10%_ had the highest tensile strength (42.41 MPa), followed by CMC/CNC_10%_ (32.22 MPa), while the lowest was observed in pure CMC (13.57 MPa). Additives typically interact with film-forming substrates through hydrogen bonds, which can enhance the tensile strength of the film [46]. Tarrés et al. [47] reported that CNF significantly improved the strength of paper due to its high surface area. The carboxylate and carboxymethyl groups in dense and layered fibers formed more hydrogen bonds between nanofibers, thereby increasing the tensile strength.

In this study, it was found that the tensile strength and elongation at the break of the CMC-based films were enhanced with the incorporation of O-CMCh and HTCMCh. Previous reports have attributed this increase to the biocompatibility, strong electrostatic interactions, and hydrogen bonding between O-CMCh and CMC [41]. On the other hand, the addition of HACC, CNC, and CNF resulted in a decrease in the elongation at break of the CMC-based films. This decrease can be attributed to the hydrogen bonds between the additive and the film-forming substrates, limiting their relative sliding possibility, thus reducing the elongation at break.

Young’s modulus is a typical characteristic of material stiffness, with a larger Young’s modulus indicating less deformability [48]. The incorporation of O-CMCh and HTCMCh in CMC-based films led to a lower Young’s modulus, indicating better toughness, superior processability, and wider applicability. The CMC/CNF film exhibited a tensile strength of 42.41 MPa and Young’s modulus of 121.17 MPa, which were 13.57 MPa and 30.84 MPa higher than those of the pure CMC film, respectively. These findings suggest that the CMC/CNF film possessed superior mechanical properties and deformation resistance. Similar results were observed in CMC/HACC films [49], CMC/CNC films [50], and graphene oxide/carboxymethyl cellulose/alginate composite blend films [51].

### 2.7. Biodegradability of CMC-Based Films

**Figure 6:**
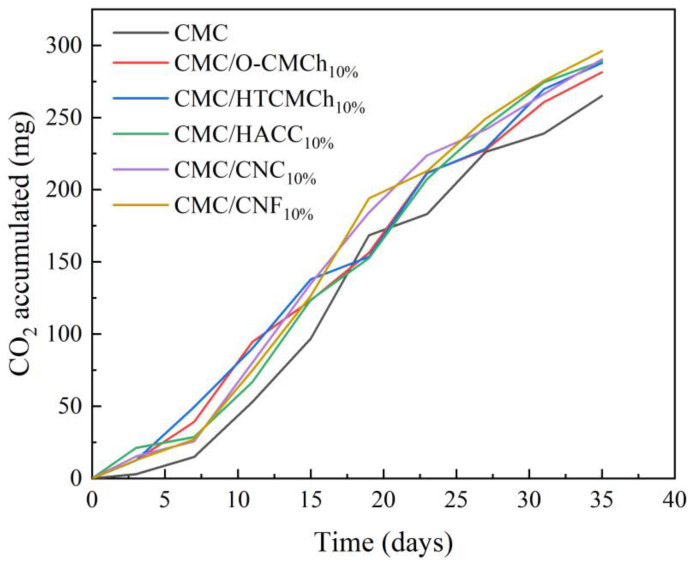
Biodegradability in terms of CO_2_ accumulation in CMC-based films.

These films were produced by incorporating chitosan and cellulose derivatives into CMC, making them suitable for various applications such as food packaging and drug delivery. However, it is crucial for these films to be biodegradable to maintain their usability. Therefore, the biodegradability of the pure CMC film and CMC film containing chitosan and cellulose derivatives was evaluated (Figure 6). To enhance the biodegradability of CMC, natural plasticizers were added, including bio-based polylactic acid (PLA) as a control to compare the level of biodegradability with other known biodegradable materials [52,53]. After a testing period of 35 days, the accumulated CO_2_ (mg) values were determined as follows: 264.92 for CMC film, 281.45 for CMC/O-CMCh film, 287.78 for CMC/HTCMCh film, 289.08 for CMC/HACC film, 290.46 for CMC/CNC, 296.12 for CMC/CNF, and 108.83 for PLA. These results demonstrate that after 35 days, some of our films had already undergone more biodegradation than PLA. The findings suggest that the biodegradability of our films is optimal, supporting their use as a replacement for non-biodegradable and environmentally harmful plastics that can have negative effects on both health and the economy.

### 2.8. Cytotoxicity of CMC-Based Films

**Figure 7:**
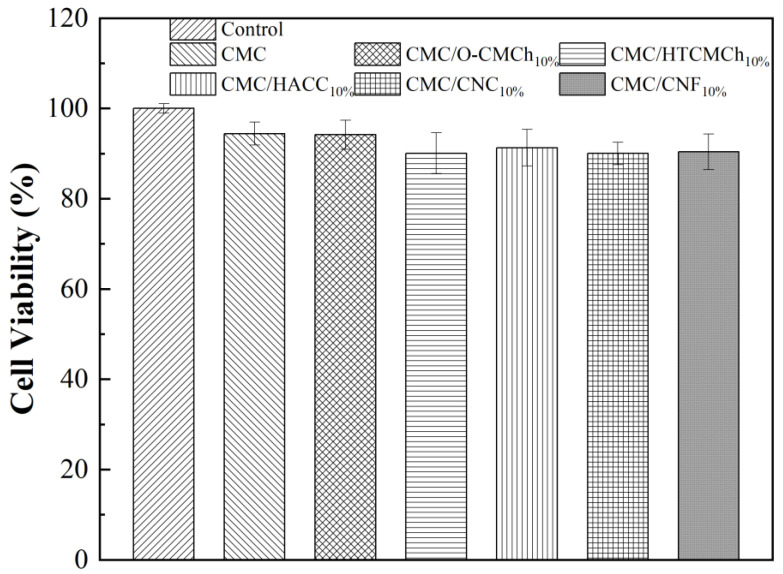
In vitro cytotoxicity of CMC-based films.

When considering the application of CMC-based films in food packaging, it is essential to assess their biocompatibility. The in vitro cytotoxicity of CMC-based films was determined by an MTT assay, with a concentration range of 0 (control sample) to 2.0 mg/mL. As shown in Figure 7, the cell viability on all of the films was above 90%, indicating that all CMC-based films were non-toxic and independent of the type of additive used. This result is consistent with previous reports [54,55,56], confirming the safety of CMC-based films for use in food packaging materials.

## 3. Experimental Section

### 3.1. Materials

**Scheme 1:**
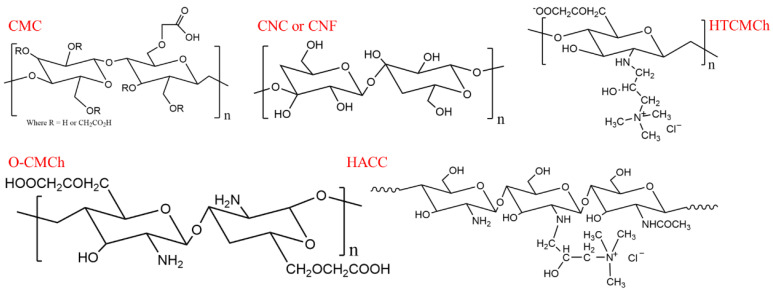
Chemical structures of the CMC, CNC, CNF, HTCMCh, O-CMCh, and HACC.

Sodium carboxymethyl cellulose (UPS grade, CMC) and O-carboxymethylated chitosan (O-CMCh) were purchased from Shanghai Aladdin Biochemical Technology Co., Ltd. (Shanghai, China). The O-CMCh had a degree of degradation ≥80% and a viscosity of 80 mPa.s. Hydroxypropyltrimethyl ammonium chloride chitosan (HACC) with a degree of substitution (DS) ≥ 98% was provided by Shanghai Macklin Biochemical Co., Ltd. (Shanghai, China). N-2-Hydroxypropyl-3-trimethylammonium-O-carboxymethyl chitosan (HTCMCh), cellulose nanofibers (CNF) with an aspect ratio of 83, and cellulose nanocrystals (CNC) with an aspect ratio of 53 were synthesized according to our previous studies [42,57,58]. The chemical structures of CMC, CNC, CNF, HTCMCH, O-CMCh, and HACC are shown in Scheme 1.

### 3.2. Preparation of Film-Forming Solutions

The preparation of the CMC/HTCMCh solution involved multiple steps. Initially, mother solutions of CMC (4% *w/v*) and HTCMCh (2% *w/v*) were prepared by magnetic stirring (600 rpm) for 4 h at 80 °C and 60 °C, respectively. These solutions were then naturally cooled to room temperature. Subsequently, a film-forming solution was created by adding specific quantities of HTCMCh and deionized water to 12.5 g of a CMC mother solution, resulting in a total weight of 25 g for the film-forming solution. The resulting solution was then subjected to magnetic stirring (600 rpm) for an additional 4 h at 60 °C before being cooled to 25 °C for future use. The mass ratios of HTCMCh to CMC (m_HTCMCh_/m_CMC_) used were 1:99, 5:95, and 10:90, and they were abbreviated as CMC/HTCMCh_1%_, CMC/HTCMCh_5%_, and CMC/HTCMCh_10%_, respectively.

Film-forming solutions of CMC/O-CMCh, CMC/HACC, CMC/CNC, and CMC/CNF were also prepared using similar abbreviations as those employed for CMC/HTCMCh.

### 3.3. Preparation of CMC-Based Films

Each film-forming solution (25 g) was poured into a polytetrafluoroethylene mold measuring 5.5 cm in diameter and 0.7 cm in height. The molds were then placed in a drying oven at 40 °C for 48 h for water evaporation.

### 3.4. Characterization of CMC-Based Films

Cross-sectional microstructures were observed on an Evo18 field-emission scanning electron microscope (FESEM) (Carl Zeiss, AG, Jena, Germany) operating at an accelerating voltage of 3.0 kV. Prior to observation, the samples were fractured using liquid nitrogen and coated with gold.

The film thickness was measured using an electronic digital Vernier caliper (Shenzhen Duliang Precision Machinery Co., Ltd., Shenzhen, China) with an accuracy of 0.001 mm. During the measurement, five random points were selected on each film and their values were averaged.

The wettability of the films was evaluated by measuring the contact angles through the sessile drop method using a KRUSS DSA 100 analyzer.

The whiteness and transmittance of the films were assessed using a YQ-Z-48B whiteness tester (Hangzhou Qingtong Brocade Automation Technology Co., Ltd., Hangzhou, China). A calibration sample of an R457 whiteboard with a whiteness of 84.5 was used. Transparency and transmittance were measured using a whiteboard with a Ry of 84% and a black background.

Thermogravimetric analysis (TGA) of the films was conducted using a thermogravimetric analyzer (Mettler Toledo, Greifensee, Switzerland) within the temperature range of 25 °C to 500 °C. The heating rate was 10 °C/min, and a nitrogen flow of 100 mL·min^−1^ was maintained.

The tensile strength (TS), elongation at break (EB), and Young’s moduli (E) were determined using an electronic universal testing machine (Jinan Teson Machinery Co. Ltd., Jinan, China). The films were cut into 4.0 × 1.0 cm strips, and the crosshead speed was set at 0.2 cm/min.

### 3.5. Rheological Measurement

The rheological properties were determined using a method previously described [18]. A DHR-2 rheometer with a parallel plate geometry of 45.0 mm in diameter and a 1.0 mm gap between the plate and sample stage was utilized. Prior to measuring the dynamic moduli, a linear viscoelastic region was identified at an angular frequency of 1.0 rad/s. The dynamic moduli, including the storage (G′) and loss moduli (G″), were then measured at angular frequencies ranging from 0.1 to 100 rad/s. The temperature was maintained at 25 °C, unless otherwise specified. The apparent viscosity was measured within a shear rate range of 0.1 to 100 s^−1^ at 25 °C.

### 3.6. Biodegradability of Films

To assess the aerobic biodegradability of the films, the UNE-EN ISO 17556 standard methodology was adopted [59]. The assay was conducted under controlled composting conditions to measure the total biodegradability of the films. A constant flow of an N_2_-O_2_ (78/23 *v*/*v*) mixture was blown into the containers with the films every 24 h, and the resulting CO_2_ was collected with a NaOH solution and subsequently titrated. After 35 days of testing, the biodegradability of each film was calculated based on the CO_2_ content.

### 3.7. In Vitro Cytotoxicity

The cytotoxicity of the CMC-based films to human foreskin fibroblast cells (HFF-1) was evaluated using the 3-(4,5-dimethylthiazol-2-yl)-2,5-diphenyltetrazolium bromide (MTT) method (Sigma, Ronkonkoma, NY, USA). Initially, the HFF-1 cells were cultured at a concentration of 104 cells/Ml in culture medium and allowed to grow for 24 h in 96-well plates. Subsequently, the HFF-1 cells were treated with 100 μL of the film-forming solution for an additional 24 h. After that, 10 μL of MTT solution (5 mg/mL) was added to each well of the 96-well plate and incubated at 37 °C for 4 h. The cells were then rinsed with phosphate-buffered saline (PBS) and dissolved in 100 μL of dimethyl sulfoxide (DMSO, 10%). The absorbance was measured at 570 nm using a microplate reader (SpectraMax M5; Molecular Devices, San Jose, CA, USA). The cell viability was calculated using the following equation (Equation (2)) [11]:

(2) $$Cell\;viability\left(\%\right)=\frac{Absorbance\;of\;treated\;sample}{Absorbance\;of\;control}\times 100$$

## 4. Conclusions

The rheological properties of CMC-based film-forming solutions were investigated, with the inclusion of additives such as O-CMCh, HTCMCh, HACC, CNC, and CNF. It was observed that all CMC-based film-forming solutions exhibited shear thinning behavior. Specifically, the solutions displayed a Newtonian plateau before reaching a shear rate of 1 s^−1^, followed by a slight shear thinning behavior. The interaction between CMC and the additives were influenced by the charge of the additives. For instance, HACC and HTCMCh primarily interacted with CMC through electrostatic interactions with some involvement of hydrogen bonds. On the one hand, O-CMCh, CNC, and CNF mainly interacted with CMC through hydrogen bonds. It was observed that chitosan derivatives had limited impact on rheological properties, whereas cellulose derivatives had a notable effect. In particular, CNF had a significant thickening effect on the CMC solutions, while CNC decreased the viscosity. The strong electrostatic attraction between HACC and CMC led to the loss of the original Newtonian plateau of CMC solutions at low shear frequencies. When the HACC content reached 5% and 10%, the CMC solutions exhibited shear thinning behavior across a shear rate range of 0.1–100 s^−l^. The rheological properties suggested good biocompatibility between CMC and all of the additives.

The films were then prepared from selected CMC-based film-forming solutions and exhibited high transmittance, hydrophilicity, improved thermal stability, enhanced tensile strength, and good biocompatibility. These results further confirmed the compatibility between CMC and the additives. The non-toxicity of the films indicated their potential for use in food packaging. The chitosan derivatives demonstrated good water solubility, and the intermolecular interaction between the chitosan derivatives and CMC in the aqueous solution was diverse. However, these interactions could not be fully revealed through the rheological experiments.

## Figures and Tables

**Table 1:** Parameters of CMC-based film-forming solutions fitted with the power-law model.

Samples	$k_{PL}$ (Pa·s)	*n*	$\eta_{1}$ (Pa·s)	$\eta_{10}$ (Pa·s)	$\eta_{100}$ (Pa·s)
CMC	6.9559	0.5321	1.9923	1.5866	0.8476
CMC/O-CMCh_1%_	7.0044	0.5243	1.9783	1.5840	0.8279
CMC/O-CMCh_5%_	6.2411	0.5459	1.7680	1.4569	0.8067
CMC/O-CMCh_10%_	6.8765	0.5391	1.9459	1.5886	0.8636
CMC/HTCMCh_1%_	6.7830	0.5249	1.8936	1.5275	0.8043
CMC/HTCMCh_5%_	7.0774	0.5316	2.0604	1.6338	0.8595
CMC/HTCMCh_10%_	6.4761	0.5434	1.8805	1.5285	0.8260
CMC/HACC_1%_	7.1250	0.5195	2.0952	1.6355	0.8237
CMC/HACC_5%_	10.5533	0.4723	4.1827	2.4424	0.9936
CMC/HACC_10%_	10.1251	0.4630	5.0657	2.4074	0.9069
CMC/CNC_1%_	5.7862	0.5361	1.8681	1.3922	0.7148
CMC/CNC_5%_	7.6036	0.5203	2.3339	1.7777	0.8841
CMC/CNC_10%_	8.8637	0.5168	2.5765	1.9835	1.0099
CMC/CNF_1%_	9.4362	0.5096	2.8358	2.1238	1.0484
CMC/CNF_5%_	8.8237	0.5054	2.6472	1.9926	0.9598
CMC/CNF_10%_	6.8930	0.5299	2.1640	1.6343	0.8308

Note: $\eta_{1}$, $\eta_{10}$, and $\eta_{100}$ represent the apparent viscosity of the CMC film-forming solution at shear rates of 1, 10, and 100 rad/s, respectively, and the R^2^ for the fitted data were all greater than 0.99.

**Table 2:** Rheological parameters of CMC-based film-forming solutions were calculated at different temperatures using the power-law model.

Samples	$k_{PL}$ (Pa·s)	*n*	$\eta_{1}$ (Pa·s)	$\eta_{10}$ (Pa·s)	$\eta_{100}$ (Pa·s)	T (°C)
CMC/O-CMCh_10%_	8.7973	0.5099	2.7071	2.0333	0.9783	15
CMC/O-CMCh_10%_	6.8765	0.5391	1.9459	1.5886	0.8636	25
CMC/O-CMCh_10%_	5.5768	0.5362	1.5683	1.2776	0.6837	35
CMC/HTCMCh_10%_	9.2297	0.5082	2.9375	2.1512	1.0171	15
CMC/HTCMCh_10%_	6.4761	0.5434	1.8805	1.5285	0.8260	25
CMC/HTCMCh_10%_	5.2879	0.5415	1.4607	1.2248	0.6657	35
CMC/HACC_10%_	11.1922	0.4578	6.2172	3.0673	1.1879	15
CMC/HACC_10%_	10.1251	0.4631	5.0656	2.4074	0.9069	25
CMC/HACC_10%_	5.6527	0.4937	2.8466	1.3937	0.5750	35
CMC/CNC_10%_	11.5919	0.4808	3.6063	2.5182	0.8147	15
CMC/CNC_10%_	8.8637	0.5168	2.5765	1.9835	1.0099	25
CMC/CNC_10%_	7.1385	0.5287	1.9328	1.6053	0.8605	35
CMC/CNF_10%_	8.0515	0.5042	2.7048	1.8902	0.8744	15
CMC/CNF_10%_	6.8930	0.5299	2.1640	1.6343	0.8308	25
CMC/CNF_10%_	5.2780	0.5327	1.5827	1.2078	0.6411	35

**Table 3:** Thickness (Th), contact angle (θ), whiteness (W), transparency (Tr), tensile strength (TS), elongation at break (EB), and Young’s modulus (E) of the CMC-based films.

Films	Th (mm)	θ (°)	W (%)	Tr (%)	TS (MPa)	EB (%)	E (MPa)
CMC	0.126 ± 0.015	44.3 ± 1.4	35.34 ± 0.13	92.72 ± 0.16	13.57 ± 0.47	44.41 ± 0.61	30.84 ± 0.96
CMC/O-CMCh_1%_	0.129 ± 0.032	50.6 ± 0.61	35.73 ± 0.08	93.63 ± 0.14	13.62 ± 0.17	49.64 ± 0.32	27.79 ± 0.53
CMC/O-CMCh_5%_	0.135 ± 0.027	47.6 ± 0.58	36.53 ± 0.16	93.67 ± 0.15	13.66 ± 0.31	54.87 ± 0.19	24.84 ± 0.89
CMC/O-CMCh_10%_	0.142 ± 0.010	40.2 ± 0.21	35.87 ± 0.18	93.33 ± 0.09	13.69 ± 0.11	58.88 ± 0.41	23.25 ± 0.98
CMC/HTCMCh_1%_	0.143 ± 0.015	46.4 ± 1.7	35.30 ± 0.36	93.42 ± 0.36	13.65 ± 0.08	60.75 ± 0.56	22.75 ± 0.68
CMC/HTCMCh_5%_	0.139 ± 0.008	54.4 ± 2.3	36.63 ± 0.41	94.76 ± 0.13	13.57 ± 0.14	62.24 ± 0.53	21.88 ± 0.65
CMC/HTCMCh_10%_	0.128 ± 0.032	57.4 ± 1.8	34.15 ± 0.42	93.65 ± 0.08	13.78 ± 0.08	64.66 ± 0.17	21.54 ± 0.64
CMC/HACC_1%_	0.148 ± 0.012	44.6 ± 0.61	35.83 ± 0.15	93.54 ± 0.17	15.65 ± 0.17	42.93 ± 0.23	36.45 ± 0.82
CMC/HACC_5%_	0.142 ± 0.018	50.3 ± 0.52	36.33 ± 0.26	93.53 ± 0.09	18.54 ± 0.13	41.72 ± 0.32	44.45 ± 0.96
CMC/HACC_10%_	0.138 ± 0.032	55.2 ± 0.45	36.20 ± 0.08	92.07 ± 0.12	20.51 ± 0.27	40.08 ± 0.03	51.17 ± 0.78
CMC/CNC_1%_	0.146 ± 0.031	46.2 ± 2.9	35.63 ± 0.36	92.16 ± 0.33	20.32 ± 0.28	43.05 ± 0.18	47.21 ± 0.77
CMC/CNC_5%_	0.152 ± 0.036	45.4 ± 1.3	35.54 ± 0.12	93.77 ± 0.05	26.76 ± 0.62	42.21 ± 0.02	63.39 ± 0.65
CMC/CNC_10%_	0.160 ± 0.019	39.3 ± 1.6	36.25 ± 0.23	91.22 ± 0.18	32.22 ± 0.37	40.81 ± 0.49	78.93 ± 0.84
CMC/CNF_1%_	0.145 ± 0.012	46.9 ± 0.9	35.54 ± 0.11	93.05 ± 0.16	28.35 ± 0.31	40.07 ± 0.13	70.75 ± 0.83
CMC/CNF_5%_	0.156 ± 0.036	43.5 ± 0.55	36.27 ± 0.13	93.77 ± 0.05	36.45 ± 0.29	37.72 ± 0.21	96.63 ± 0.96
CMC/CNF_10%_	0.161 ± 0.021	38.7 ± 0.6	36.16 ± 0.16	92.22 ± 0.28	42.41 ± 0.46	34.74 ± 0.33	121.17 ± 0.94

## Data Availability

The data presented in this study are available on request from the corresponding author.
